# Depression among Latina women in the United States from pregnancy to early postpartum: longitudinal examination of risk factors

**DOI:** 10.1186/s12884-025-07548-6

**Published:** 2025-04-25

**Authors:** Naomi V. Rodas, Sabrina R. Liu, Denise A. Chavira, Job G. Godino

**Affiliations:** 1 Laura Rodriguez Research Institute, Family Health Centers of San Diego, 1750 5th Ave, San Diego, CA 92101 USA; 2 Department of Human Development, California State University, San Marcos, CA USA; 3 Department of Psychology, University of California, Los Angeles, Los Angeles, CA USA; 4 Herbert Wertheim School of Public Health and Human Longevity Science, University of California San Diego, San Diego, CA USA

**Keywords:** Perinatal depression, Latinx, Pregnancy, Maternal mental health

## Abstract

**Background:**

The present study examined the prevalence of symptoms of depression throughout pregnancy and the early postpartum period (first trimester through six weeks postpartum) in low-income Latina women. Further, this study examined whether established risk factors in non-Latina women also predict perinatal depressive symptom trajectories in low-income Latinas.

**Methods:**

The sample included 240 Latina women from a federally qualified health center (FQHC).

**Results:**

Latent growth curve modeling found that, on average, symptoms of depression decreased from the first trimester of pregnancy through six weeks postpartum. Change in symptoms of depression from the first trimester through six weeks postpartum were predicted by limited English proficiency and age.

**Conclusions:**

Findings of the current study add to existing evidence that can guide clinical care for Latina maternal mental health throughout the perinatal period.

## Background

### Perinatal depression and latina populations

The perinatal period, defined as the period spanning from pregnancy, birth, and through the first twelve months post birth involves profound psychological and physiological transitions for women. Approximately 1 in 5 women experience symptoms of depression during the perinatal period [1], and morbidity related to perinatal depression is well-documented as the most common childbirth complication [2]. Perinatal depression has harmful effects on a mother’s functioning and well-being, and longitudinally impacts the social-emotional and developmental functioning of children [3, 4]. For example, depression is associated with increased risk of pregnancy and birth complications, preterm birth, lower infant birth weight, increased infant hospital length of stay, and long-term child cognitive and emotional deficits [1, 5].

Latinx individuals are the largest ethnic minority group in the United States [6] and Latina mothers’ fertility rate is 12% greater than the national average [7]. In California, almost 50% of all live births are to Latina women. Further, Latina women in California experience perinatal symptoms of depression almost twice as often as white women (17.1% compared to 9.5%; Maternal Child and Adolescent Health Division, 2018), yet are much less likely to access mental health treatment [1]. Thus, it is imperative to better understand and address perinatal depression within this population.

### Risk factors

Prior research has identified risk factors for perinatal depression. Age is inversely associated with depressive symptoms, with younger pregnant individuals demonstrating higher levels of symptomatology [8, 9]. Social support from one’s social network is also an important predictor of fewer depressive symptoms in the perinatal period [10, 11]. Social support encompasses formal support from health professionals and services as well as informal support from friends, family, and others in one’s social network. There are also different facets of support including emotional, instrumental (e.g., providing assistance), informational, and appraisal (e.g., communication of praise and/or respect) [11]. Last, history of mood disorder symptoms and stress during the prenatal period are also risk factors for recurring symptomatology during the perinatal period [8, 12].

Specific to Latina women, Lara-Cinisomo and colleagues outlined a biopsychosocial framework for risk of postpartum depression which encompasses contextual (i.e., poverty and traumatic experiences) as well as cultural (i.e., acculturative stress and discrimination) pathways of risk of psychopathology [13]. In support of this model, a recent study found that discrimination and domestic violence predicted higher levels of early postpartum depressive symptoms in a sample of low-income Latina mothers [14]. Additionally, language preference, which has been demonstrated to be a brief proxy of acculturation [15], has also been examined as a risk factor for postpartum depression in Latinas. Some studies have demonstrated English language preference to be associated with increased risk of postpartum depression [16, 17], while other studies have shown language preference to be unrelated to risk of postpartum depression [14, 18]. Important next steps for advancing understanding of perinatal depression risk among Latina women is examining the context-specific stressors outlined in Lara-Cinisomo’s framework throughout pregnancy.

### Longitudinal research on perinatal depression

It is also important to examine trajectories of depression throughout the perinatal period to better inform perinatal depression screening and targeted prevention and intervention efforts. To date, there remains vast heterogeneity in research findings related to perinatal depressive symptoms, symptom severity, and trajectories [8, 19–21]. Some studies have found that depression symptoms tend to be higher early on in pregnancy and decrease into the postpartum period [22], while others have found evidence for distinct trajectories, including a low-to-no symptom trajectory (i.e., low or no symptoms during the perinatal period), a high symptom episodic trajectory (i.e., temporarily increased depressive symptoms during pregnancy or the postpartum period), and a high-decreasing trajectory (i.e., initially high levels of depressive symptoms which decrease throughout the perinatal period; [19, 21].

This research has contributed important understanding of the heterogeneity of perinatal depression over time, but it is overwhelmingly based on samples of non-Hispanic white women from middle to high socioeconomic backgrounds. This is despite lower-income and racial and ethnic minority pregnant women having higher birth rates and being disproportionately more likely to experience chronic stress and perinatal depression [23, 24]. Mora and colleagues examined trajectories of depressive symptoms from pregnancy through two years postpartum among a large sample of low-income, multiethnic women and found evidence for five distinct trajectories: always or chronic depressive symptomatology (7% of their sample); 2) antepartum only (6% of their sample); 3) postpartum, resolving after one year (9% of the sample); 4) late, present at 25 months postpartum (7% of the sample); and 5) never having depressive symptomatology (71% of the sample). Latina women (17% of the sample) were less likely to belong to the trajectories of antepartum or postpartum depression than white women [25]. Another large study of low-income women found evidence for low, high, increasing, and decreasing trajectories of depression from the first trimester through six months postpartum. In contrast to Mora and colleagues, they did not find Latina women (25% of the sample) were more or less likely to belong to one trajectory or another compared to women of other races/ethnicities [26]. Given the limited research depression trajectories specifically among Latina women, this is an important area for further investigation to inform best practices for perinatal depression symptom screening and intervention within this population [27].

### Current study

The current study aimed to characterize depression symptoms throughout pregnancy and the early postpartum period (first trimester, second trimester, third trimester, and fourth trimester) among 240 Latina women from a federally qualified health center (FQHC) in Southern California. Further, this study examined whether culturally and contextually-specific stressors outlined in Lara-Cinisomo’s biopsychosocial framework (history of domestic violence, language) and risk factors documented in non-Latinx women populations (age, education, income, mental health history, social support) predict perinatal depressive symptom trajectory. Based on prior research, it was predicted that age, education, income, and social support would be inversely related to depressive symptoms. It was also expected that mental health history and history of domestic violence would positively predict depressive symptoms. Lastly, women whose primary language was English were predicted to be less symptomatic than women whose primary language was not English. We did not have any hypotheses regarding symptom trajectory and sought to examine these in an exploratory fashion.

## Methods

### Participants and study design

Participants were 240 women receiving perinatal healthcare within a large Federally Qualified Health Center’s (FQHC’s) prenatal support services department in Southern California. All participants included in the study completed a Broad Informed Consent, which includes specific authorization for the use of de-identified health information for population health studies. Patient care data were de-identified for this purpose. Women were eligible for inclusion in this study if their pregnancy overlapped with the study period, they attended at least two Prenatal Social Work appointments including the first trimester appointment and identified as Latino/Hispanic.

The study was approved by the Institutional Review Board at the host institution. A retrospective chart review was conducted, covering from January 2022 through July 2023. The chart review included demographic information (mother’s race/ethnicity, preferred language, age, level of education, household income), Edinburgh Postnatal Depression Scale (EPDS) scores, and data from the initial psychosocial assessment conducted. Like other longitudinal studies across pregnancy and postpartum, there were 25 women that did not have complete data for the EPDS during the second trimester, 105 women that did not have complete data for the EPDS during the third trimester, and 144 that did not have complete data for the EPDS at six weeks postpartum.

### Measures

*Edinburgh Postnatal Depression Scale.* The Edinburgh Postnatal Depression Scale (EPDS; 26) was utilized to assess perinatal depression during the first, second, third, and fourth trimesters of pregnancy. The EPDS is a validated questionnaire used to assess symptoms of depression and anxiety in perinatal populations. It is a ten-item self-report questionnaire that asks participants to rate each item on how they felt over the past seven days. Scores range from 0 to 30, with higher scores indicating greater levels of symptomatology. The EPDS has been shown to have cross-ethnic/racial measurement equivalence [28, 29].

*Psychosocial Assessment.* The Comprehensive Perinatal Services Program Psychosocial Assessment was used by social workers to gather demographic, psychosocial, mental health history, and information pertaining to the women’s pregnancy. Psychosocial assessments were conducted at each trimester throughout pregnancy and once during the postpartum period by bachelor’s level social workers. Mental health history was assessed by asking the following Yes/No question, “Have You Had Problems With Depression Or Other Mental Illness?”. History of domestic violence was measured by asking the following Yes/No question, “Have you ever been emotionally or physically abused by your partner or someone important to you?”. Social support was measured by taking the sum of the following dichotomous question items: “How does the father of the baby feel about the pregnancy?” with options being “Supportive/Involved” versus “Not Supportive”; “How does your family feel about the pregnancy?” with options being “Supportive/Involved” versus “Not Supportive”; and “How do your friends feel about the pregnancy?” with options being “Supportive/Involved” versus “Not Supportive”. Information regarding women’s age, primary language (English versus Spanish), and annual household income was also gathered.

### Data analytic plan

We employed latent growth curve modeling (LGCM; i.e., growth models estimated within the Structural Equation Model framework) utilizing Mplus 8.0 [30], to examine the developmental trajectory of perinatal mood symptoms (first trimester through six weeks postpartum) and to examine the individual effects of our key variables measured during the first trimester (age, education, income, language, mental health history, history of domestic violence, and social support) in predicting first trimester symptoms of perinatal depression and change in perinatal depression symptoms. LGCM allows for examining individual differences in change over time, as well as examining what factors are associated with these changes [31]. In LGCM, repeated measures of the outcome construct (i.e., depressive symptoms) serve as indicators of latent growth factors. Participants’ reports of perinatal depression symptoms on the EPDS at first trimester, second trimester, third trimester, and during their postpartum visit were used as indicators to estimate latent factors (intercept and slope). The intercept factor was centered at the first trimester. The linear slope factor represented the rate of change in EPDS scores (first trimester through postpartum). Next, predictors were added into the model and continuous predictors were centered at the grand mean. We utilized the comparative fit index (CFI; values at or above 0.95 indicate adequate fit) and the root-mean-square error of approximation (RMSEA; values at or below 0.05 = excellent fit, 0.05–0.09 = good fit, and over 0.10 = inadequate fit) [32]. Models were estimated using the full information maximum likelihood (FIML) estimator, which allows the inclusion of participants with only partial data present. Full Information Maximum Likelihood has been demonstrated to be a robust estimator [33, 34], leading to less biased estimates for coefficients and standard errors when compared to utilizing listwise deletion.

## Results

### Participant characteristics

Table 1 presents notable participant characteristics. On average, women were 28 years old during their first trimester visits (*M* = 28.93, SD = 5.39). Less than half of the sample (34%) had an education beyond high school, and more than two-thirds (82%) of the sample had a household income of less than $30,000. Notably, 39% of the sample indicated they had no household income ($0). Most subjects (91%) were partnered, including living with their partners and/or married. One-fifth of the sample endorsed current or past mental health problems. 48% of the sample endorsed Spanish as their primary language, while 52% endorsed English as their primary language. Last, 13% of the sample indicated current or past experiences of physical or emotional abuse.

**Table 1:** Participant characteristics

Characteristic	Percent (*n*)
Maternal Education Level	
High school or less	65.83% (158)
More than high school	34.17% (82)
Income	
$0	25.00% (60)
$1-$9,999	8.75% (21)
$10,000-$19,999	24.17% (58)
$20,000-$29,999	24.17% (58)
$30,000-$39,999	10.42% (25)
$40,000-$49,999	5.00% (12)
$50,000-$99,999	2.50% (6)
Relationship Status	
Non-partnered	8.55% (21)
Partnered	91.45% (219)
Experienced emotional or physical abuse	12.92% (31)
Current or past mental health problems	18.33% (44)

Table 2 displays participants’ depression levels as measured by the EPDS. EPDS scores were highest on average in the first trimester (*M* = 3.54) and lowest in the third trimester (*M =* 2.45). Similarly, the greatest proportion of women (8.75%) scored in the possible-to-probable depression range in the first trimester, followed by the second trimester (8.37%), postpartum (7.37%), and third trimester (5.18%).

**Table 2:** The Edinburgh postnatal depression scale (EPDS) score ranges

	First Trimester *N* = 240	Second Trimester *N* = 215	Third Trimester *N* = 135	6 Weeks Postpartum *N* = 95
EPDS score (*M*,* SD*)	3.54 (4.24)	2.93 (3.66)	2.45 (3.20)	2.88 (3.69)
Possible depression (%)	2.50%	5.58%	3.70%	4.21%
Probable depression (%)	6.25%	2.79%	1.48%	3.16%

Note. Possible depression indicates percent of Edinburgh Postnatal Depression Scale (EPDS) between 10 and 12; probable depression indicates percent of EPDS scores greater than 12

### LGCM results

*Change Over Time.* The unconditional (without covariates) model indicated that symptoms of perinatal depression on average decreased from the first trimester of pregnancy to the fourth trimester (postpartum). A negative linear slope was found to best capture change in symptoms of depression across the perinatal period (Intercept: *B* = 3.41, *SE* = 0.26, *p* <.001; Slope: *B* = -0.33, *SE* = 0.12, *p* <.01). The unconditional model was determined to have good fit with the data, *X*^2^ = 9.22 [5], *p* =.10, CFI = 0.97, RMSEA = 0.06.

Next, to examine the effect of age, language, education, income, mental health history, history of domestic violence, and social support on symptoms of perinatal depression over time, we fit a conditional model, and these factors were included as predictors. The final model was determined to have excellent fit with the data, *X*^2^ = 15.97 [19], *p* =.65, CFI = 1.00, RMSEA = 0.00.

*Individual Effects.* Language, mental health history, and history of domestic violence predicted initial levels of symptoms of perinatal depression (Table 3). Women who spoke Spanish as their primary language presented with higher levels of perinatal depression during the first trimester compared to women whose primary language was English. Women who endorsed previously having experienced difficulties with mental health symptoms also presented with higher levels of depressive symptoms during the first trimester compared to those who did not. Last, women who endorsed being a victim previously of domestic violence presented with higher levels of depressive symptoms compared to those who did not endorse such experiences. The intercept was not predicted by age, education level, income, nor social support.

Change over time (linear slope) was predicted by language and age. On average, the change rate in symptoms of perinatal depression from first trimester through six weeks postpartum was higher for Latina women whose primary language was English. Therefore, English-speaking women’s perinatal depression symptoms decreased at a faster rate throughout the perinatal period compared to non-English speaking women. Moreover, older age was associated with a steeper decrease in depressive symptoms for women over time.

**Table 3:** Individual effects latent growth curve model

	Intercept at First Trimester	Linear Slope 1st -4th Trimesters
**Variable**	**B (SE)**	**B (SE)**
Age	-0.03 (0.04)	0.05** (0.02)
Language	-1.09* (0.49)	0.47* (0.23)
Education	-0.57 (0.52)	0.20 (0.24)
Income	0.00 (0.49)	-0.45 (0.23)
Mental Health History	3.08*** (0.68)	-0.44 (0.32)
History of Domestic Violence	1.83* (0.79)	-0.08 (0.37)
Social Support	-0.05(0.17)	-0.01 (0.08)

*p <.05, **p <.01, ***p <.001

## Discussion

The present study examined the effects of culturally and contextually specific stressors (i.e., history of domestic violence and language) in addition to age, education, income, mental health history, and social support on first trimester symptoms of perinatal depression and change in perinatal depression symptoms from first trimester through six weeks postpartum of pregnancy among a sample of Latina women seeking care at a FQHC. We used latent growth curve modeling and found that, on average, symptoms of perinatal depression decreased from the first trimester through six weeks postpartum. This aligns with results from prior studies that have also found depression symptoms to be higher early on in pregnancy and decrease throughout the perinatal period in other non-Latina populations [22]. In another study, Wenzel and colleagues found that Latina women had a unique trajectory of anxiety symptoms compared to non-Hispanic black women in that their anxiety symptoms were highest early in pregnancy and then decreased [35]. There was also a trend in the same direction for depressive symptoms (Wenzel et al., 2021). Results of the current study suggest a similar pattern for prenatal depressive symptoms among Latina women. It may be the case that women’s mood is most highly impacted early on in pregnancy as they are adjusting to the new transitional role of being pregnant. It could also be the case that symptoms may be decreasing as a result of accessing perinatal and mental health care.

Initial levels of perinatal depression during the first trimester of pregnancy were positively predicted by history of mental health and history of domestic violence. This is aligned with findings of other studies [8, 12]. Ponting and colleagues (2020) found domestic violence experienced during pregnancy and early postpartum to be associated with higher levels of postpartum depression in Latinas [14]. Our study expands upon these findings by examining the effects of prior experiences of domestic violence on depressive symptoms during pregnancy and into the early postpartum period. Findings from this study illustrate the long-term impact interpersonal violence can have within this population of women in particular. Our research adds to existing research that has documented domestic violence history as a predictor of postpartum depressive symptoms [13, 14], showing that domestic violence is also a predictor for depression in the prenatal period. Experiencing intimate partner violence can erode a woman’s self-esteem and sense of identity and increase feelings of despair, guilt, and loneliness [36], all of which may increase vulnerability to depression particularly during the prenatal period, when women are coping with the increased physiological and emotional stress of pregnancy.

Additionally, Latinas who reported Spanish as their primary language reported more symptoms of perinatal depression compared to those whose primary language was English. This differs from Ponting and colleagues (2020) findings that language preference was not associated with symptoms of postpartum depression. Our study, however, examined symptomatology during the first trimester of pregnancy. Spanish speaking Latinas, who are likely less acculturated, may encounter increased difficulties navigating new systems of health care [37]. It may be the case that language barriers are particularly impactful to Latinas’ mental health when first coming into regular contact with a large medical system.

Next, we examined the rate of change in symptoms of perinatal depression from the first trimester through the fourth trimester and found that it was predicted by language and age. Symptoms of perinatal depression decreased at a faster rate for English-speaking Latinas compared to Spanish speaking Latinas. This is in contrast to existing studies that have found either no association between language preference and perinatal depression, or preference for English and higher levels of perinatal depression [16, 38, 39]. This disparity may be related to greater difficulty accessing services due to language barriers, highlighting the importance of accessible, bilingual, bicultural perinatal mental health services. Additionally, given that language has often been found to be a proxy for acculturation, it may be the case that less acculturated Latinas’ mental health may be negatively impacted by the various compounding life transitions (e.g., adapting to life in the U.S. while also adapting to transitions involved in pregnancy). Prior literature has also demonstrated that Latina women with low health literacy are more likely to suffer from depression during pregnancy and postpartum (for review see Kilfoyle et al., 2016) [40]. Therefore, health literacy may be a salient risk factor that could be targeted for Spanish speaking Latinas throughout pregnancy to mitigate the effect on symptoms of depression.

Further, increasing age was associated with a decrease in perinatal depression over time. This is consistent with prior studies examining perinatal depression in other ethnic groups of women [9]. This is likely to reflect the challenges that arise with being a young parent, as well as perhaps the difficulty adjusting to this new role throughout the perinatal period. This could have also been for reasons related to resources and support, as older women are more likely to have better-developed emotion regulation capacities, independent living skills, support systems, and higher job satisfaction and income [41–44].

### Limitations & future directions

While results of our study provide important insights to better understanding predictors and trajectories of perinatal depressive symptoms among low-income Latina women, our findings cannot necessarily be applied broadly across the United States. The Latina population in the United States is diverse, therefore our results should be considered in context with the specific characteristics of our sample. Our sample comes from a single geographic area in Southern California [13]. Additionally, in contrast with previous studies [10, 11], we did not find associations between social support and depressive symptoms. Our measure of social support was limited to assessing perceived support from baby’s father, mother’s family, and friends in a yes/no manner. The measure did not assess level of support on a Likert scale or in different domains, including emotional support, instrumental support, informational support, and appraisal support (Sufredini et al., 2022). Future research could benefit from utilizing more comprehensive measures of social support to examine their association with perinatal depressive symptoms over time. We also acknowledge the need to replicate our findings utilizing a larger sample size of Latina women. A larger sample size would also allow for the examination of the possibility of multiple distinct trajectories that could then be compared to the findings of others who have identified several trajectories in low-income, multiethnic samples [25, 26].

## Conclusions

Findings of the current study add to existing evidence that can guide clinical care. Knowledge and understanding of perinatal mental health disorders among the general public is lacking, particularly discussions of mental health disorders in the prenatal period. However, our findings suggest that the prenatal period, and particularly the first trimester, may be the time of highest risk for depressive symptoms among low-income Latina women. Therefore, early assessment and intervention of depressive symptoms is a critical component of prenatal care, particularly for this population. Care providers may want to take note of presence or absence of the risk factors identified in this study, namely prior mental health problems and trauma history, and trauma-informed care should be incorporated into mental health treatment. Our results also indicate that younger women may need more mental health support, as their symptoms did not decrease over time as much as older women in our sample. Future research should continue to examine targets of intervention for Latinas throughout the perinatal period, as well as tailor existing evidence-based treatments based on these findings.

## Data Availability

Due to the nature of this research, participants of this study did not agree for their data to be shared publicly, so supporting data is not available. The corresponding author can be contacted if someone would like to request the data from this study.

## Abbreviations

FQHC: Federally qualified health center
EPDS: Edinburgh postnatal depression scale
LGCM: Latent growth curve modeling
CFI: Comparative fit index
RMSEA: Root-mean-square error of approximation
FIML: Full information maximum likelihood
